# Convection-Enhanced Delivery of Tumor-Infiltrating Lymphocytes Enhances Intratumoral Distribution and Therapeutic Efficacy in an Orthotopic Rat Glioma Model

**DOI:** 10.3390/biomedicines14071466

**Published:** 2026-06-28

**Authors:** Yuan Zhou, Liwen Zhu, Xinglei Liu, Chunxia Ji, Jiakai Yao, Di Chen, Yu Yao

**Affiliations:** 1 Department of Neurosurgery, Huashan Hospital, Fudan University, 958# Jinguang Road, Shanghai 200000, China; yuanzhou22@m.fudan.edu.cn (Y.Z.); levinezhu@163.com (L.Z.); laliuxing@163.com (X.L.); 13211010007@fudan.edu.cn (C.J.); 2 National Center for Neurological Disorders, Fudan University, Shanghai 200000, China; 3 Immunology Laboratory, Neurosurgical Institute of Fudan University, Fudan University, Shanghai 200000, China; zh946818@163.com

**Keywords:** glioblastoma, convection-enhanced delivery, tumor-infiltrating lymphocytes, adoptive cell therapy, immunotherapy

## Abstract

**Background:** Adoptive cell therapy using tumor-infiltrating lymphocytes (TILs) is a potential strategy for glioma treatment, but effective intracranial delivery remains a major obstacle. Convection-enhanced delivery (CED) may improve local parenchymal coverage by bypassing the blood–brain barrier and using pressure-driven interstitial transport. **Methods:** We evaluated whether CED could improve the early intracerebral distribution and antitumor activity of *ex vivo*-expanded TILs in an orthotopic rat C6 glioma model. Expanded TILs were characterized as a CD3-enriched lymphocyte product with inducible effector function against C6 glioma cells *in vitro*. TILs were administered as either Control-TILs by Hamilton syringe-based conventional intratumoral injection or CED-TILs by catheter-based CED infusion using matched cell dose, volume, infusion rate, target coordinates, and dwell time. Intracerebral CD3^+^ T-cell coverage, tumor progression, and overall survival were assessed. Short-term safety was evaluated in a separate cohort of naïve rats receiving CED-PBS or CED-TILs. **Results:** CED-TILs produced broader early intraparenchymal CD3^+^ T-cell coverage than Control-TILs, particularly at distal sampling sites from the infusion tract. Under this single-dose regimen, CED-TILs were associated with reduced tumor progression, decreased Ki67 expression, increased apoptosis-associated signaling, and prolonged survival. In the short-term naïve safety cohort, CED-TILs did not produce overt neurologic, histologic, hematologic, or systemic toxicity within the observation window. **Conclusions:** These findings support CED-TILs as an early proof-of-concept locoregional delivery strategy that improves early spatial CD3^+^ T-cell coverage and is associated with antitumor activity in a rat glioma model.

## 1. Introduction

Glioblastoma (GBM) represents the most common and lethal primary intracranial malignancy in adults. Despite the current standard of care, including maximal surgical resection followed by radiotherapy and temozolomide chemotherapy, the prognosis remains dismal, with a median survival of less than 15 months [1]. In recent years, immunotherapy, particularly adoptive cell therapy (ACT) using tumor-infiltrating lymphocytes (TILs), has demonstrated remarkable efficacy in solid tumors such as metastatic melanoma [2]. However, the application of ACT in brain tumors faces unique physiological hurdles.

The primary obstacle restricting the efficacy of systemic T-cell therapy in gliomas is the blood–brain barrier (BBB) and related neurovascular interfaces, which limit immune-cell trafficking and penetration into the tumor parenchyma [3]. Furthermore, elevated interstitial fluid pressure (IFP) and abnormal fluid transport within solid tumors, including brain tumors, can generate pressure/flow conditions that impede convective transport and limit therapeutic penetration [4,5]. Consequently, intravenously administered lymphocytes often fail to infiltrate the tumor core in sufficient numbers to induce a robust antitumor response.

To overcome these barriers, convection-enhanced delivery (CED) has emerged as a locoregional delivery strategy in which controlled positive-pressure micro-infusion drives therapeutics through the brain interstitium via a stereotaxically placed catheter [6,7]. Importantly, clinical CED implementations use carefully titrated flow rates, reflux-mitigating catheter designs, and imaging/physiologic monitoring to balance distribution with safety in eloquent brain regions [8]. CED complements other BBB-bypass approaches, including focused ultrasound (FUS)-mediated BBB opening and intrathecal/intraventricular delivery, each with distinct advantages and limitations for achieving uniform intraparenchymal exposure. Despite expanding interest in cellular immunotherapies, data regarding the feasibility, spatial distribution, and safety of CED for delivering cellular therapeutics such as TILs remain limited [9].

In this study, we tested whether CED-TILs could improve the local spatial coverage of adoptively transferred TILs in the brain compared with Control-TILs and whether this delivery advantage was associated with antitumor effects in an orthotopic rat glioma model. We established a standardized stereotaxic CED workflow, benchmarked CED-TILs against Control-TILs, quantified early intracerebral CD3^+^ T-cell distribution and tumor readouts, and performed a separate short-term safety assessment under matched infusion parameters. Together, these experiments aim to define the feasibility and key practical considerations of CED-enabled intracranial ACT while highlighting the remaining questions required for clinical translation.

## 2. Materials and Methods

### 2.1. Study Design, Animals and Ethics

All animal procedures complied with the ARRIVE guidelines and were approved by the Institutional Animal Care and Use Committee of Huashan Hospital, Fudan University (protocol #202003004S). Female Sprague-Dawley rats (≈6 weeks old, 150–180 g) were housed under SPF conditions (12 h light/dark cycle; 22–24 °C) with food and water ad libitum. Animals were randomized using computer-generated sequences, and outcome assessments (histology/quantification and behavioral tests) were performed by investigators blinded to group allocation.

### 2.2. Experimental Cohorts and Sample Size

To improve transparency and reproducibility, experiments were organized into predefined animal cohorts. Unless otherwise specified, each animal contributed a single independent data point (biological replicate).

Cohort 1 (tumor-bearing efficacy): C6-bearing rats were randomized to NC, Control-TILs (Hamilton syringe-based conventional intratumoral injection of TILs), or CED-TILs (catheter-based CED infusion of TILs). Overall survival was predefined as the primary efficacy endpoint. Longitudinal bioluminescence imaging (BLI) was used as a secondary tumor-burden endpoint to monitor tumor progression over time. Group sizes were NC (*n* = 8), Control-TILs (*n* = 8), and CED-TILs (*n* = 8). Survival was defined as the time from tumor implantation to death or euthanasia at a predefined humane endpoint.

Cohort 2 (tumor-bearing tissue analyses): A separate set of randomized C6-bearing rats underwent the same treatments and was harvested at predefined early time points for CD3 IHC distribution mapping (Day 1) and for Ki67 IF and western blotting (Day 3).

Group sizes and the exact tissue endpoints per animal are detailed in the corresponding figure legends and in Supplementary Table S1.

Cohort 3 (naïve safety): Non-tumor-bearing rats received intracerebral CED-PBS or CED-TILs using the same catheter and infusion parameters as in the tumor-bearing cohorts. Acute neurobehavioral testing, CBC, organ weights, histology, and TEM were performed at the indicated time points. Group sizes were CED-PBS (*n* = 8) and CED-TILs (*n* = 8).

### 2.3. Orthotopic C6 Glioma Model

Rats were anesthetized with isoflurane (5% induction, 2% maintenance) and positioned in a stereotaxic frame. C6 glioma cells (provided by Huashan Hospital, Fudan University; 5 × 10^6^ cells in 10 µL PBS) were injected into the right striatum at AP +1.0 mm, ML +2.0 mm, DV −3.5 mm (relative to bregma) using a 50 µL Hamilton syringe at 1.0 µL/min. The needle was kept in place for 10 min to minimize reflux before slow withdrawal.

### 2.4. TIL Isolation, CD3 Enrichment, and Expansion

Tumor tissue for TIL isolation was harvested from donor rats bearing orthotopic C6 gliomas at Day 10 post-implantation (tumor confirmed by bioluminescence imaging and gross inspection). For each expansion, tumors from 2–3 donor rats were pooled to yield approximately 200–300 mg of tissue. Fresh glioma tissue was minced and enzymatically digested using collagenase IV (Worthington Biochemical, Lakewood, NJ, USA; Cat. No. LS004188) and DNase I (Grade II, Roche, Basel, Switzerland; Cat. No. 10104159001) at 37 °C for 30 min. After filtration through a 70 µm cell strainer, leukocytes were enriched by Percoll density gradient centrifugation (30%/70%) (Cytiva, Marlborough, MA, USA; Cat. No. 17-0891-01). CD3^+^ T cells were enriched by magnetic separation using Pan T Cell MicroBeads, rat (Miltenyi Biotec, Bergisch Gladbach, Germany; Cat. No. 130-090-320) according to the manufacturer’s instructions. Purified TILs were expanded in RPMI-1640 supplemented with 10% heat-inactivated FBS (Gibco, Thermo Fisher Scientific, Grand Island, NY, USA; Cat. No. 16140071), penicillin-streptomycin, L-glutamine, and recombinant rat IL-2 (PeproTech, Cranbury, NJ, USA; Cat. No. 400-02-20UG) at 300 IU/mL for 10–12 days with medium refreshed every 2–3 days. For functional assays (ELISpot/co-culture/RTCA), expanded TILs were washed to remove residual IL-2 and rested overnight (12–16 h) in IL-2–free medium before stimulation as described below.

### 2.5. Flow Cytometry Quality Control of CD3-Enriched TILs

As product quality control (QC) prior to intracranial administration, expanded TILs were assessed by flow cytometry for viability and CD3/CD4/CD8 composition. Cells were stained with Fixable Viability Dye eFluor™ 780 (eBioscience, Thermo Fisher Scientific, Waltham, MA, USA; Cat. No. 65-0865-14) and antibodies against rat CD45, CD3ε-FITC (clone 1F4; BD Pharmingen, BD Biosciences, San Jose, CA, USA; Cat. No. 561801), CD4-PE (clone W3/25; BD Pharmingen, BD Biosciences, San Jose, CA, USA; Cat. No. 572406), and CD8α-APC (clone OX-8; eBioscience, Thermo Fisher Scientific, Waltham, MA, USA; Cat. No. 17-0084-82). Data were acquired on a NovoCyte flow cytometer (Agilent Technologies, Santa Clara, CA, USA) and analyzed using FlowJo v10.8.1. The gating strategy was: singlets → live cells → CD45+ leukocytes → CD3+ T cells, followed by CD4/CD8 subset composition.

### 2.6. TIL Stimulation Conditions for Functional Assays

For antigen-recall stimulation, bone marrow-derived dendritic cells (DCs) were generated from naïve rats using a standard GM-CSF/IL-4 protocol. Briefly, bone marrow cells were cultured with recombinant rat GM-CSF (PeproTech, Cranbury, NJ, USA; Cat. No. 400-23-20UG; 20 ng/mL) and recombinant rat IL-4 (PeproTech, Cranbury, NJ, USA; Cat. No. 400-04-20UG; 10 ng/mL) for 6 days, and matured with lipopolysaccharide from *Escherichia coli* O111 (Sigma-Aldrich, St. Louis, MO, USA; Cat. No. L2630; 100 ng/mL) overnight. DCs were pulsed with C6 tumor lysate (DC/C6-Lys; 50 μg/mL total protein) for 4 h, washed, and co-cultured with rested TILs at a DC ratio of 1:10. For polyclonal activation, TILs were stimulated with plate-bound anti-rat CD3 antibody (αCD3; clone eBioG4.18/G4.18; eBioscience, Thermo Fisher Scientific, Waltham, MA, USA; Cat. No. 16-0030-85) at 1 μg/mL. Unstimulated TILs (rested in IL-2–free medium) served as baseline controls. The same stimulation conditions were applied for ELISpot, bright-field co-culture, and RTCA unless otherwise specified.

### 2.7. IFN-γ ELISpot

Rat IFN-γ ELISpot (Mabtech, Nacka Strand, Sweden; Cat. No. 3221-4APW-2) was performed according to the manufacturer’s protocol. Rested TILs were plated in triplicate and left unstimulated or stimulated as described above (DC/C6-Lys or αCD3) for 18–24 h. Plates were read on an ImmunoSpot^®^ S5 analyzer (Cellular Technology Limited, Shaker Heights, OH, USA).

### 2.8. Real-Time Cytotoxicity Assay (RTCA)

For RTCA, C6 cells were seeded in E-Plate 16 plates (Agilent Technologies, Santa Clara, CA, USA; Cat. No. 300600890; 8 × 10^3^ cells/well). After stabilization for 24 h, rested TILs were added at a predefined effector-to-target ratio (E = 2:1) under the indicated stimulation conditions, including unstimulated TILs, TILs stimulated with C6 lysate-pulsed dendritic cells (DC/C6-Lys), or polyclonally activated TILs (αCD3). Electrical impedance, expressed as cell index (CI), was recorded every 5 min for up to 48 h using the Agilent xCELLigence RTCA S16 system (Agilent Technologies, Santa Clara, CA, USA).

For analysis and visualization, CI values were normalized within each well to the last measurement immediately prior to T-cell addition to generate the normalized cell index (NCI = CI/CI(*t0*)), and time was aligned to the T-cell addition time point (*t* = 0). Unless otherwise stated, TILs used in functional assays were the CD3-enriched expanded product without additional FACS sorting.

Each condition was tested in triplicate technical wells using the same expanded TIL preparation. These replicate wells were used to assess intra-assay technical reproducibility and were not considered independent TIL donor preparations or independent expansion batches. Batch-to-batch variability among independent TIL expansions was not systematically assessed in the present study.

### 2.9. Bright-Field Co-Culture and Confluence Quantification

For bright-field co-culture, C6 cells were seeded in 96-well plates (1 × 10^4^ cells/well). After adherence, TILs were added at the indicated effector-to-target (E:T) ratios (2:1). Images were acquired at 0, 24, and 48 h. Tumor cell confluence was quantified as a percentage of area covered by adherent C6 cells using consistent thresholding parameters across groups/time points.

### 2.10. Intracranial TIL Delivery: Control-TILs Versus CED-TILs

Rats bearing orthotopic gliomas were allocated to three groups: NC (no treatment), Control-TILs (Hamilton syringe-based conventional intratumoral injection of TILs), and CED-TILs (catheter-based CED infusion of TILs). All intracranial administrations were performed under isoflurane anesthesia using the same stereotaxic coordinates as tumor implantation (AP +1.0 mm, ML +2.0 mm, DV −3.5 mm relative to bregma).

To minimize procedural confounding between Control-TILs and CED-TILs, the two TIL delivery procedures were matched for stereotaxic trajectory, target depth, TIL dose, infusion volume, infusion rate, dwell time, and withdrawal procedure. Specifically, both groups received 1 × 10^7^ TILs suspended in 20 μL of PBS at 2 μL/min, followed by a 5 min dwell period before slow withdrawal of the injection needle or catheter. Thus, the main intended experimental difference between the two groups was the delivery hardware and the associated pressure-driven convection field.

For Control-TILs, TILs were delivered by Hamilton syringe-based conventional intratumoral injection using a 50 μL Hamilton syringe (Hamilton Company, Reno, NV, USA). For CED-TILs, an RWD 27G brain catheter (RWD Life Science, Shenzhen, China, ID/OD 0.11/0.21 mm; tip length 1.0 mm) connected to a micro-infusion pump (SpLab02; Baoding Shenchen Precision Pump Co., Ltd., Baoding, Hebei, China) was used to deliver the same cell dose and volume under pump-controlled CED infusion conditions. The injection needle or catheter was retained for 5 min after infusion and then slowly withdrawn to minimize reflux.

Tumor implantation was performed separately using a smaller volume and slower injection rate (5 × 10^6^ C6 cells in 10 μL PBS at 1 μL/min) to optimize reproducible tumor establishment. Therapeutic TIL delivery used 20 μL of cell suspension to maximize local coverage. The Control-TIL procedure was therefore designed to match dose, volume, flow rate, dwell time, and stereotaxic coordinates with the CED-TIL condition, while differing primarily in the delivery hardware and pressure-driven convection mechanism.

For distribution mapping, brains were harvested on post-treatment Day 1 for CD3 immunohistochemistry. For tumor biology readouts, a separate set of treated animals was harvested on post-treatment Day 3 for Ki67 immunofluorescence and western blotting of cleaved PARP, cleaved caspase-3, and Ki67.

### 2.11. Infusion Monitoring and Success Criteria

During each infusion, the tubing and catheter were inspected for air bubbles, leakage, or obstruction. Pump operation and completion of the planned infusion volume were confirmed for each animal. Gross reflux was monitored visually at the burr hole and cortical surface when possible during infusion and catheter withdrawal. Infusion was considered successful when the catheter reached the predefined stereotaxic target, the full planned volume was delivered without visible tubing leakage or catheter obstruction, no marked gross reflux was observed, the 5 min dwell period was completed, and the animal recovered from anesthesia. Prespecified exclusion criteria included incomplete infusion, catheter obstruction, tubing leakage, marked reflux, incorrect catheter placement, major procedure-related hemorrhage, or procedure-related death before endpoint. Infusion pressure was not directly recorded because the current system did not include an inline pressure sensor.

### 2.12. CD3 Immunohistochemistry and Spatial Sampling

Brains were harvested on post-treatment Day 1 for CD3 immunohistochemistry, perfused with PBS followed by 4% paraformaldehyde, paraffin-embedded, and sectioned at 5 μm. After citrate antigen retrieval (pH 6.0), sections were incubated with anti-CD3 antibody (Abcam, Cambridge, UK; Cat. No. ab5690), followed by HRP/DAB detection and hematoxylin counterstaining.

To evaluate early spatial CD3^+^ T-cell coverage while minimizing *z*-axis sampling bias, serial coronal sections spanning the entire infusion/needle tract were collected using a systematic sampling scheme (every 200 μm; total 6 sections per brain). For each section, predefined regions of interest (ROIs) of identical size were placed at 0, 2, and 4 mm radially from the tract center along a consistent anatomical axis. Distribution was operationally defined as CD3^+^ cell density at these predefined radial distances from the infusion/needle tract. ROI placement was performed according to predefined anatomical rules and avoided necrotic areas, tissue folds, large vessels, ventricles, hemorrhagic regions, and mechanically damaged tissue.

Images were acquired under identical microscope settings within the same staining batch, including objective magnification, illumination intensity, camera exposure, and white-balance settings. Background DAB signal was assessed using adjacent CD3-negative tissue regions, and the same thresholding criteria were applied across all groups. CD3^+^ cells were defined as DAB-positive nucleated cells within each ROI and were reported as CD3^+^ T cells per mm^2^. Quantification was performed by investigators blinded to the treatment group. For each animal, values were first averaged across ROIs and serial sections to yield one representative value per animal at each distance.

### 2.13. Ki67 Immunofluorescence and Quantification

Brains were harvested on post-treatment Day 3 for Ki67 immunofluorescence analysis. Paraffin-embedded tumor-bearing brain sections were deparaffinized, rehydrated, subjected to citrate antigen retrieval, blocked, and incubated with anti-Ki67 antibody (Abcam, Cambridge, UK; Cat. No. ab16667), followed by staining with Alexa Fluor^®^ 594-conjugated goat anti-rabbit IgG secondary antibody (Abcam, Cambridge, UK; Cat. No. ab150080) and DAPI nuclear counterstaining (Beyotime Biotechnology, Shanghai, China; Cat. No. C1006).

Ki67 positivity was quantified as the percentage of Ki67^+^ nuclei among total DAPI^+^ nuclei within predefined tumor-region ROIs. Tumor boundaries were identified on the same section or adjacent serial sections based on H&E morphology, DAPI nuclear density, and tumor-associated tissue architecture. Fixed-size ROIs were placed at matched anatomical locations relative to the infusion/needle tract and the tumor margin, with emphasis on the peri-tract tumor–parenchyma interface. Necrotic regions, hemorrhagic areas, tissue folds, large vessels, ventricles, and mechanically damaged tissue were excluded from analysis.

Images were acquired using identical microscope settings within the same staining batch, including objective magnification, illumination intensity, exposure time, gain, and channel settings. Background fluorescence was estimated from adjacent Ki67-negative tissue regions and subtracted uniformly. DAPI^+^ nuclei and Ki67^+^ nuclei were identified using the same thresholding criteria across all groups. Quantification was performed using ImageJ software v1.54f (National Institutes of Health, Bethesda, MD, USA) by investigators blinded to the treatment group. For each animal, values from multiple ROIs across systematically sampled sections were averaged to generate one representative Ki67 positivity value per animal.

### 2.14. Western Blotting and Densitometry

Tumor tissues were dissected on post-treatment Day 3 from Cohort 2 animals, separate from the survival-monitoring cohort, snap-frozen, and homogenized in RIPA lysis buffer (Beyotime Biotechnology, Shanghai, China; Cat. No. P0013B) supplemented with PMSF (Beyotime Biotechnology, Shanghai, China; Cat. No. ST506). Protein concentrations were determined using a BCA protein assay (Beyotime Biotechnology, Shanghai, China; Cat. No. P0012). Equal amounts of total protein (20 μg per lane) were separated by SDS-PAGE and transferred to PVDF membranes (Millipore, Burlington, MA, USA; Cat. No. IPVH00010). After blocking, membranes were incubated with primary antibodies against cleaved PARP (Cell Signaling Technology, Danvers, MA, USA; Cat. No. 5625), cleaved caspase-3 (Cell Signaling Technology, Danvers, MA, USA; Cat. No. 9664), Ki67 (Abcam, Cambridge, UK; Cat. No. ab16667), and β-actin (Abcam, Cambridge, UK; Cat. No. ab8227;), followed by HRP-linked anti-rabbit IgG secondary antibody (Cell Signaling Technology, Danvers, MA, USA; Cat. No. 7074) and chemiluminescent detection using BeyoECL Plus reagent (Beyotime Biotechnology, Shanghai, China; Cat. No. P0018S).

Chemiluminescent images were acquired under non-saturating exposure conditions. Images with saturated bands were excluded from densitometric analysis, and only exposure images in which the analyzed bands were visually unsaturated and within the linear detection range were used for quantification. Band intensities were quantified using ImageJ software v1.54f (National Institutes of Health, Bethesda, MD, USA). Target protein signals were first normalized to β-actin from the same lane, and normalized values were then expressed relative to the mean value of the NC group.

### 2.15. Tumor Burden and Survival

#### 2.15.1. Bioluminescence Imaging (BLI)

For BLI, rats received D-luciferin ((GoldBio, St. Louis, MO, USA; Cat. No. LUCK-1G; 150 mg/kg, i.p.)) and were imaged using an IVIS Spectrum system (PerkinElmer, Waltham, MA, USA; 60 s exposure, binning 8, f/1) with identical acquisition settings across animals and time points. Images were acquired at a fixed interval after luciferin injection. Tumor-associated BLI signal was quantified using a standardized ROI-based pipeline in Living Image software v4.5.4 (PerkinElmer, Waltham, MA, USA). A fixed cranial ROI encompassing the tumor-bearing hemisphere was applied consistently to each animal across time points, and background signal was measured from an equivalently sized non-tumor region and subtracted from the tumor ROI. BLI signal was quantified as total flux (photons/s). For longitudinal comparison, post-treatment BLI values were normalized to the pre-treatment baseline value of the same animal. ROI placement and BLI quantification were performed using identical templates by investigators blinded to the treatment group.

#### 2.15.2. Survival Analysis

Overall survival was defined as the time from tumor implantation to death or euthanasia at a predefined humane endpoint. Animals were monitored daily by investigators blinded to group allocation. Humane endpoints included >20% body-weight loss, severe or progressive neurological deficits, inability to eat or drink, moribund status, or other signs of severe distress. Animals that died from procedure-unrelated causes or were removed from the study for non-tumor-related reasons before reaching the endpoint were censored at the last day known alive. No animals were censored for tumor-related endpoints. Survival curves were generated using the Kaplan–Meier method and compared using the log-rank test.

### 2.16. Safety Evaluation in Naïve Rats

For TEM, peri-tract cortex (~1–2 mm) was collected on Day 3, processed by standard fixation/embedding, and imaged at 80 kV (JEOL Ltd., Akishima, Tokyo, Japan). Hematology was measured on Days 1, 3, and 7 using Sysmex XS-500i (Sysmex Corporation, Kobe, Hyogo, Japan). Body weight was recorded on Days 0/1/3/7. Sensorimotor function was assessed by the cylinder test and whisker-evoked forelimb placing. On Day 7, the heart, liver, spleen, lungs, kidneys, and brain were weighed.

### 2.17. Statistics

All analyses were performed using GraphPad Prism v10 (GraphPad Software, Boston, MA, USA) and R v4.3.0 (R Foundation for Statistical Computing, Vienna, Austria). Data are presented as mean ± SD (or mean ± SEM as specified). For multi-group comparisons, one-way ANOVA or two-way ANOVA with appropriate multiple-comparisons correction was applied as indicated in the figure legends. Survival was compared using the log-rank (Mantel–Cox) test; hazard ratios (HR) with 95% confidence intervals are reported where applicable. A two-sided *p* < 0.05 was considered statistically significant. Sample sizes for each experiment are reported in the figure legends and Supplementary Table S1. No a priori power calculation was performed; sample sizes were chosen based on prior C6 survival studies and practical constraints, and should be interpreted accordingly.

## 3. Result

### 3.1. Results 1: Expanded Rat TILs Exhibit a Stable CD3^+^ Phenotype and Stimulus-Dependent Effector Function In Vitro

To support reproducible intracranial delivery and experimental transparency, we first established a standardized CED workflow integrating a micro-infusion pump with stereotaxic guidance (Figure S1a). A 27G brain infusion catheter (ID/OD 0.11/0.21 mm; tip length 1.0 mm) was used for catheter-based CED infusion (Figure S1b), and the surgical procedure was standardized stepwise, including hair removal, disinfection, scalp incision, burr-hole drilling, cannula placement, and wound closure (Figure S1c). The overall experimental design was organized into three predefined cohorts: tumor-bearing efficacy assessment, tumor-bearing tissue analysis, and naïve safety evaluation (Figure S1e).

**Figure 1:**
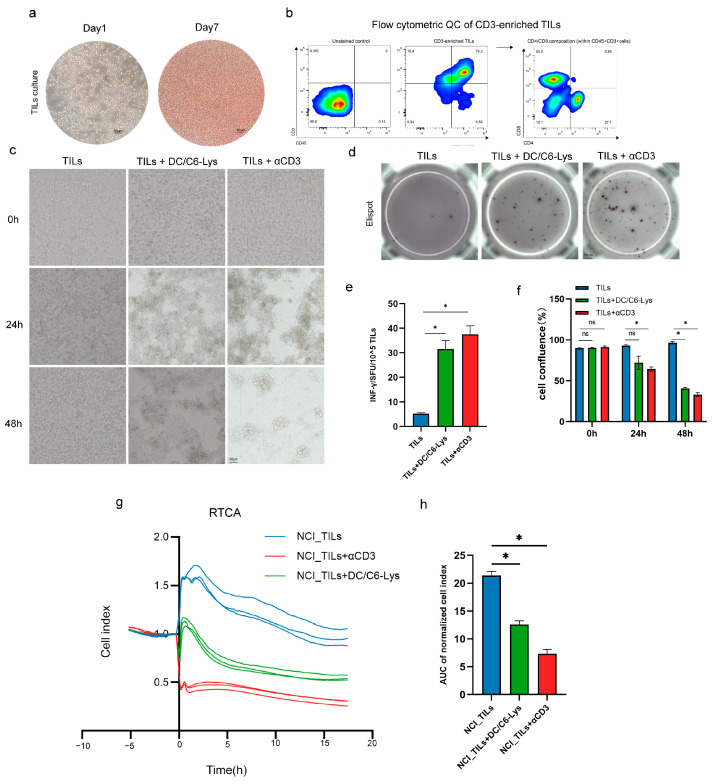
Phenotypic and functional characterization of expanded rat TILs *in vitro***.** (**a**), Representative phase-contrast images of rat TIL cultures on Day 1 and Day 7 after *ex vivo* expansion. Scale bar, 50 μm. (**b**), Representative flow-cytometric quality control of CD3-enriched TILs. Left, unstained control; middle, CD3^+^ T-cell gating within CD45^+^ leukocytes; right, CD4/CD8 subset composition within live CD45^+^CD3^+^ T cells. Numbers indicate the percentage of gated cells. (**c**), Representative bright-field images of C6 glioma cells co-cultured with expanded TILs under the indicated stimulation conditions: unstimulated TILs, TILs stimulated with C6 lysate-pulsed dendritic cells (TILs + DC/C6-Lys), and polyclonally activated TILs (TILs + αCD3). Images were acquired at 0, 24, and 48 h after co-culture. (**d**), Representative IFN-γ ELISpot wells from TILs under the same stimulation conditions as in (**c**). (**e**), Quantification of IFN-γ ELISpot responses, expressed as spot-forming units (SFU) per 10^5^ TILs. (**f**), Quantification of adherent C6 tumor-cell confluence (%) in the bright-field co-culture assay at 0, 24, and 48 h. (**g**), Real-time cell analysis (RTCA) of C6 cells co-cultured with TILs under the indicated conditions. Impedance is presented as normalized cell index (NCI), calculated within each well as CI/CI(*t_0_*), where t0 represents the last measurement immediately before TIL addition. Time is plotted relative to TIL addition (*t* = 0). (**h**). Quantification of RTCA cytotoxicity using the area under the normalized cell index curve (AUC of NCI) from 0 to 18 h after TIL addition. For ELISpot, bright-field co-culture, and RTCA assays, *n* = 3 technical replicate wells per condition from the same expanded TIL preparation, not independent TIL donor preparations. Data in bar graphs are presented as mean ± SD. Statistical comparisons among three groups were performed using the Kruskal–Wallis test followed by Dunn’s multiple-comparison test. For tumor-cell confluence in (**f**), comparisons were performed separately at each time point. ns, not significant; * *p* < 0.05.

Following *ex vivo* expansion, rat TIL cultures formed dense cellular aggregates over time, as shown by representative phase-contrast images from Day 1 and Day 7 (Figure 1a). Flow-cytometric quality control confirmed that the expanded product was predominantly composed of CD3^+^ leukocytes containing both CD8^+^ and CD4^+^ subsets (Figure 1b). The gating strategy included debris exclusion, singlet gating, live-cell gating using eFluor 780, and subsequent CD45^+^CD3^+^ T-cell and CD4/CD8 subset analysis (Figure S1f), supporting the use of the expanded product for downstream functional and intracranial delivery assays.

Functionally, IFN-γ ELISpot showed low basal activity in unstimulated TILs, whereas stimulation with C6 lysate-pulsed dendritic cells (TILs + DC/C6-Lys) or polyclonal activation with anti-CD3 (TILs + αCD3) increased IFN-γ spot formation (Figure 1d,e), consistent with inducible effector potential. In bright-field co-culture with C6 glioma cells, unstimulated TILs showed limited cytotoxicity, whereas TILs stimulated by DC/C6-Lys or αCD3 induced progressive tumor-cell clearance over 24–48 h (Figure 1c,f). Consistently, impedance-based RTCA demonstrated a decline in normalized cell index (NCI) after addition of stimulated TILs, with the strongest suppression observed in the αCD3 condition and an intermediate effect in the DC/C6-Lys condition compared with unstimulated TILs (Figure 1g). Quantification of the RTCA response using the AUC of NCI from 0 to 18 h further supported enhanced cytotoxic activity after stimulation (Figure 1h). Together, these data indicate that the expanded CD3-enriched TIL product retained stimulus-dependent effector function against C6 glioma cells *in vitro*.

### 3.2. Results 2: CED Delivery Broadens Intracerebral T-Cell Distribution and Enhances Antitumor Effects in Orthotopic Glioma

In the orthotopic C6 glioma model, rats were assigned to NC, Control-TILs, or CED-TILs groups according to the predefined efficacy and tissue-analysis cohorts (Figure S1e). Control-TILs were administered by Hamilton syringe-based conventional intratumoral injection, whereas CED-TILs were administered by catheter-based CED infusion. To minimize procedural confounding, both TIL-treated groups received the same total cell dose, infusion volume, infusion rate, stereotaxic target, and dwell time.

**Figure 2:**
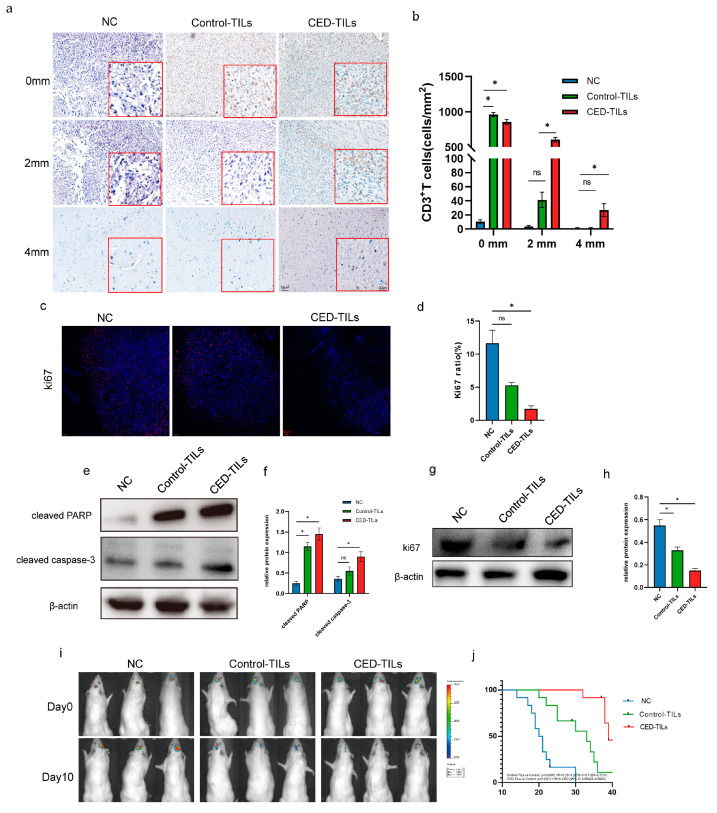
CED improves intracerebral T-cell distribution and enhances antitumor efficacy in an orthotopic rat glioma model. (**a**) Representative CD3 immunohistochemistry (IHC) images showing early spatial CD3^+^ T-cell coverage in NC, Control-TILs, and CED-TILs groups. Control-TILs were administered by Hamilton syringe-based conventional intratumoral injection, whereas CED-TILs were administered by catheter-based convection-enhanced delivery. Control-TILs and CED-TILs received the same TIL dose, infusion volume, infusion rate, stereotaxic target, and dwell time: 1 × 10^7^ TILs in 20 μL PBS at 2 μL/min, followed by a 5 min dwell period before slow withdrawal. Brains were harvested on post-treatment Day 1. Sections were sampled at 0, 2, and 4 mm radially from the infusion/needle tract. Red boxes indicate representative ROIs used for CD3^+^ cell quantification. Scale bar, 50 μm. (**b**) Quantification of CD3^+^ T-cell density at each radial distance, expressed as CD3^+^ cells per mm^2^. (**c**) Representative Ki67 immunofluorescence images of intracranial tumors from NC, Control-TILs, and CED-TILs groups. Tumor tissues were collected on post-treatment Day 3. (**d**) Quantification of Ki67 positivity, expressed as the percentage of Ki67^+^ nuclei among total DAPI^+^ nuclei within predefined tumor-region ROIs. (**e**), Representative western blots of apoptosis-associated markers, including cleaved PARP and cleaved caspase-3, with β-actin as the loading control. Tumor tissues were collected on post-treatment Day 3. (**f**) Densitometric quantification of cleaved PARP and cleaved caspase-3 in (**e**), shown as relative protein expression normalized to β-actin. (**g**) Representative western blot of Ki67 with β-actin as the loading control. (**h**) Densitometric quantification of Ki67 in (**g**) shown as relative protein expression normalized to β-actin. (**i**) Representative longitudinal bioluminescence imaging (BLI) of intracranial tumors in NC, Control-TILs, and CED-TILs groups at baseline and follow-up. BLI signals were acquired using identical imaging settings and quantified as total flux within a fixed cranial ROI after background subtraction, with longitudinal values normalized to the pre-treatment baseline signal of the same animal. (**j**) Kaplan–Meier survival curves of rats in the indicated groups. Overall survival was predefined as the primary efficacy endpoint. Data are presented as mean ± SD unless otherwise indicated. For CD3 IHC, Ki67 IF, and western blot quantification, *n* = 3 animals per group, with each animal contributing one averaged value from predefined ROIs and/or sampled sections. For survival analysis, *n* = 8 animals per group. Multi-group comparisons were performed using the Kruskal–Wallis test followed by Dunn’s multiple-comparison test. Survival curves were compared using the log-rank test. ns, not significant; * *p* < 0.05.

On post-treatment Day 1, CD3 immunohistochemistry demonstrated sparse CD3^+^ signal in NC animals, whereas both TIL-treated groups showed prominent peri-tract/peritumoral CD3^+^ signals at the 0 mm sampling site (Figure 2a). Compared with Control-TILs, CED-TILs maintained higher CD3^+^ T-cell density at distal sampling sites, with detectable CD3^+^ cells at 2 mm and 4 mm from the infusion/needle tract (Figure 2a,b). Quantification across predefined radial distances confirmed broader early radial CD3^+^ T-cell coverage in the CED-TILs group relative to Control-TILs (Figure 2b). Because CD3 staining alone cannot distinguish infused TILs from endogenous T cells recruited secondarily, these findings are interpreted as increased early CD3^+^ T-cell coverage rather than definitive tracking of the infused product.

Tumor-biological readouts were assessed on post-treatment Day 3. Ki67 immunofluorescence showed reduced Ki67 positivity after TIL treatment, with the lowest Ki67 signal observed in the CED-TILs group (Figure 2c,d). Western blotting further showed increased apoptosis-associated markers, including cleaved PARP and cleaved caspase-3, in TIL-treated tumors, with the strongest increase observed in the CED-TILs group (Figure 2e,f). In parallel, Ki67 protein expression was reduced in TIL-treated tumors, again most prominently in the CED-TILs group (Figure 2g,h). These data suggest that broader early CD3^+^ T-cell coverage after CED-TILs was associated with reduced proliferation-associated signals and increased apoptosis-associated signaling in tumor tissue.

Longitudinal bioluminescence imaging showed lower tumor-associated photon signals in the CED-TILs group compared with NC and Control-TILs at the examined time points (Figure 2i). As the predefined primary efficacy endpoint, overall survival was compared among the NC, Control-TILs, and CED-TILs groups. Kaplan–Meier analysis showed a survival benefit after TIL treatment, with the greatest prolongation observed in the CED-TILs cohort (Figure 2j). Collectively, these results support an early proof-of-concept interpretation that catheter-based CED-TILs improved early intracerebral CD3^+^ T-cell coverage and were associated with antitumor activity under the tested single-dose conditions.

### 3.3. Results 3: Short-Term Safety Profile of Intracerebral CED-TILs in Naïve Rats

**Figure 3:**
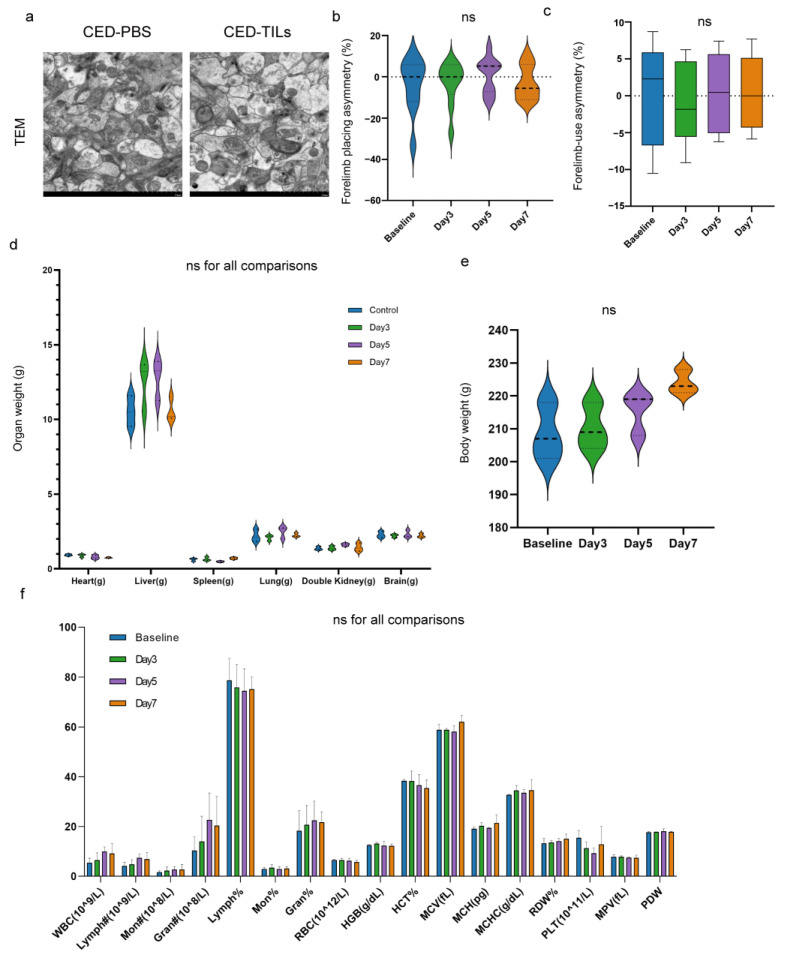
Short-term safety evaluation of CED-PBS and CED-TILs in naïve rats. (**a**) Representative transmission electron microscopy (TEM) images of peri-infusion cortex from naïve rats receiving CED-PBS or CED-TILs, showing preserved neuronal and axonal ultrastructure within the short-term observation window. CED-TILs were delivered using the same infusion parameters as in the tumor-bearing efficacy experiments: 1 × 10^7^ TILs suspended in 20 μL PBS at 2 μL/min, followed by a 5 min dwell period before slow catheter withdrawal. CED-PBS animals received the same vehicle volume using the same CED hardware and infusion procedure. Scale bar, 1 μm. (**b**) Whisker-evoked forelimb placing asymmetry (%) measured at baseline before infusion and on post-infusion Days 3, 5, and 7. Violin plots show the distribution of values; center lines indicate the median with quartiles. (**c**), Forelimb-use asymmetry (%) in the cylinder test measured at baseline and on post-infusion Days 3, 5, and 7. (**d**) Organ weights of heart, liver, spleen, lung, paired kidneys, and brain measured at the indicated time points. (**e**) Body weight measured at baseline and on post-infusion Days 3, 5, and 7. Violin plots show the distribution of values; center lines indicate the median with quartiles. (**f**) Complete blood count (CBC) parameters measured at baseline and on post-infusion Days 3, 5, and 7. Baseline refers to pre-infusion measurements obtained before intracerebral CED. Data are presented as median with quartiles for violin plots and as mean ± SD for bar graphs. For longitudinal repeated measurements in the same animals, statistical comparisons across time points were performed using the Friedman test followed by Dunn’s multiple-comparison test. For independent endpoint comparisons, the Kruskal–Wallis test followed by Dunn’s multiple-comparison test was used. No overt short-term neurologic, hematologic, body-weight, organ-weight, ultrastructural, or systemic toxicity was detected under the tested infusion parameters. ns, not significant.

To evaluate procedure- and product-related tolerability independent of tumor burden, naïve rats received intracerebral CED-PBS or CED-TILs using the same CED hardware, catheter system, and infusion parameters as in the tumor-bearing experiments (Figure S1a–c,e). Transmission electron microscopy of peri-infusion cortex showed preserved neuronal and axonal ultrastructure in the CED-TILs group, without obvious myelin disruption or cytopathologic abnormalities compared with CED-PBS controls within the short-term observation window (Figure 3a).

Neurologic function was assessed longitudinally using whisker-evoked forelimb placing and cylinder tests. Forelimb placing asymmetry and forelimb-use asymmetry did not show overt treatment-associated deficits after infusion (Figure 3b,c). Body weight remained stable over the observation period (Figure 3e), and organ weights did not show apparent abnormalities across the examined time points (Figure 3d). Complete blood count parameters remained within expected ranges after infusion, with no obvious hematologic abnormalities attributable to CED-TILs under the tested conditions (Figure 3f). Representative H&E sections of major organs, including heart, liver, spleen, lung, and kidney, showed no overt histopathologic abnormalities after CED-TILs compared with CED-PBS controls (Figure S1d).

Together, these short-term safety readouts did not reveal overt neurologic, ultrastructural, hematologic, body-weight, organ-weight, or systemic toxicity after intracerebral CED-TILs in naïve rats under the current infusion parameters. However, these findings should be interpreted as short-term tolerability data in non-tumor-bearing animals and do not exclude delayed neuroinflammation, tumor-bearing brain toxicity, repeated-dose toxicity, or late off-target effects.

## 4. Conclusions

In summary, our study provides early proof-of-concept evidence that CED-TILs can improve locoregional delivery of expanded TILs into the brain parenchyma in an orthotopic rat glioma model. Compared with Control-TILs, CED-TILs produced broader early CD3^+^ T-cell spatial coverage away from the infusion tract and were associated with reduced tumor proliferation, increased apoptosis-associated signaling, lower longitudinal bioluminescence signals, and prolonged survival under the tested single-dose conditions.

In a matched short-term safety cohort of non-tumor-bearing rats, evaluations including transmission electron microscopy of peri-infusion cortex, hematology, body weight, sensorimotor testing, organ weights, and histopathology did not reveal overt procedure- or product-related toxicity within the observation window. Together, these findings support CED-TILs as a feasible preclinical locoregional delivery approach for improving early intraparenchymal T-cell coverage in malignant glioma models. However, further studies are required to optimize TIL product characteristics, dose, infusion parameters, catheter design, and treatment schedule; to evaluate long-term persistence, trafficking, functional state, repeated-dose safety, delayed neuroinflammation, edema, off-target damage, and tumor-bearing safety; and to test combination regimens in more clinically representative and immunocompetent models before clinical translation can be considered.

## 5. Discussion

In this study, we evaluated convection-enhanced delivery (CED)-mediated infusion of expanded tumor-infiltrating lymphocytes (TILs) as an early delivery-focused proof-of-concept strategy for intracranial adoptive cell therapy in an orthotopic rat glioma model. Under the tested single-dose conditions, CED-TILs improved early intraparenchymal CD3^+^ T-cell coverage and were associated with reduced tumor progression and prolonged survival compared with Control-TILs. Glioblastoma remains one of the most lethal primary brain tumors despite maximal surgical resection followed by radiotherapy and temozolomide-based chemotherapy [1]. Adoptive cell therapy (ACT), particularly TIL therapy, has achieved meaningful clinical activity in selected extracranial solid tumors, most notably melanoma [2,10]. However, translating T-cell-based therapies to glioblastoma remains challenging because of the blood–brain barrier (BBB), abnormal tumor-associated fluid dynamics, low baseline lymphocyte infiltration, antigenic heterogeneity, and the highly immunosuppressive tumor microenvironment [3,5,11,12,13,14]. Recent discussions of glioma immunotherapy and related preclinical models have also emphasized the need for clinically relevant models, clear immune-cell product characterization, and rigorous functional readouts when evaluating new glioma immunotherapy strategies [14]. The current study addresses one specific component of this challenge: the physical delivery barrier that limits local immune-cell coverage within intracranial tumors.

Previous studies have suggested that TILs can be isolated and expanded from glioma specimens, supporting their potential as a cellular source for malignant glioma immunotherapy [15]. Early clinical experience with local administration of autologous TILs or tumor-specific T cells in recurrent malignant gliomas also suggested feasibility and acceptable tolerability, although antitumor efficacy was variable and often limited by delivery, persistence, and immune escape [16]. Compared with these earlier approaches, the present study focused specifically on whether CED could improve the early local distribution of TIL-associated CD3^+^ cells within brain tumor tissue. This question is clinically relevant because inadequate cellular penetration and heterogeneous intratumoral distribution may prevent ACT from achieving sufficient effector-to-target contact across invasive glioma regions.

A central finding of our study was that CED-TILs produced broader early intracerebral CD3^+^ T-cell coverage than Control-TILs. At the 0 mm sampling site, both Control-TILs and CED-TILs generated robust CD3^+^ signals, whereas at 2 and 4 mm from the infusion/needle tract, CED-TILs maintained higher CD3^+^ T-cell density. This distribution pattern is consistent with the core principle of CED, in which a positive-pressure gradient supplements diffusion and drives bulk flow through the brain interstitium [17]. CED has been widely explored for the delivery of macromolecules, toxins, nanoparticles, viral vectors, and immunomodulatory agents in brain tumors [6,7,8,9,18]. Our data extend this delivery concept to adoptive cellular therapy by showing that, under matched dose and volume conditions, the delivery route can influence early spatial CD3^+^ T-cell coverage.

However, this distribution result requires cautious interpretation. First, CD3 immunostaining alone cannot distinguish infused TILs from endogenous T cells that may have been secondarily recruited to the tumor microenvironment after treatment. Therefore, the distal CD3^+^ signal observed after CED-TILs may reflect a combination of physically dispersed infused TILs, retained T cells, and host immune-cell recruitment. Future studies using fluorescent dye labeling with appropriate controls, genetically labeled donor cells, infused-cell tags, sex-mismatch PCR, or single-cell RNA/TCR profiling will be required to directly determine the persistence, trafficking, and spatial fate of infused TILs after intracerebral delivery. Second, our spatial analysis was based on predefined radial distances from the infusion/needle tract rather than whole-section grid-based mapping, three-dimensional reconstruction, or volume-of-distribution/infused-volume (Vd/Vi) analysis. Thus, the current data demonstrate improved early radial CD3^+^ T-cell coverage, but do not provide a complete volumetric characterization of cell distribution across the tumor-bearing hemisphere.

The improved early CD3^+^ T-cell coverage was accompanied by measurable antitumor readouts. Ki67 immunofluorescence and western blotting showed reduced proliferation-associated signals after TIL treatment, with the greatest reduction in the CED-TILs group. In parallel, cleaved PARP and cleaved caspase-3 were increased, indicating enhanced apoptosis-associated signaling. These findings are consistent with the in vitro functional assays, in which expanded TILs retained inducible effector function, including IFN-γ secretion and cytotoxic activity against C6 glioma cells under antigen-recall or polyclonal stimulation conditions. Together, these results suggest that improving the physical coverage of TIL-associated CD3^+^ cells within the brain tumor microenvironment can be associated with downstream changes in tumor biology. Nevertheless, these data should be interpreted as early antitumor activity under the tested conditions rather than definitive evidence of durable therapeutic efficacy.

The infused cell product also requires cautious interpretation. TIL therapy differs from CAR-T therapy in that it may contain polyclonal tumor-reactive T-cell populations rather than a single engineered specificity. CAR-T cell studies targeting antigens such as EGFRvIII or IL13Rα2 have demonstrated that cellular therapies can be manufactured and delivered to patients with glioblastoma, and that local or regional administration may generate intracranial immune activity [19,20]. However, these studies also highlight persistent challenges, including antigen heterogeneity, adaptive immune resistance, limited persistence, and the suppressive glioma microenvironment [19,21,22]. In the present study, we used a bulk-expanded CD3-enriched TIL product with inducible effector function, but we did not deeply characterize tumor-reactive clonotypes, antigen specificity, exhaustion markers, differentiation state, memory phenotype, stem-like versus terminally differentiated phenotypes, polyfunctional cytokine profiles, or TCR diversity. Therefore, the current study cannot determine whether the observed therapeutic activity was driven by a specific TIL subset or whether CED preferentially improved the delivery, retention, or function of particular T-cell populations. Future studies should incorporate markers such as PD-1, TIM-3, LAG-3, TOX, CD137/4-1BB, CD44, CD62L, CCR7, CD127, Ki67, granzyme B, perforin, IFN-γ/TNF-α/IL-2 polyfunctionality, and T-cell receptor sequencing or single-cell RNA/TCR sequencing.

The comparison between Control-TILs and CED-TILs is central to this study. To minimize procedural confounding, the two TIL-treated groups were matched for total TIL dose, infusion volume, infusion rate, stereotaxic target, dwell time, and withdrawal procedure. Specifically, both groups received 1 × 10^7^ TILs suspended in 20 μL PBS at 2 μL/min, followed by a 5 min dwell period before slow withdrawal. These parameters were not independently optimized for each route; the main experimental difference was the delivery hardware and the associated pressure-driven convection field. Even with matched parameters, however, route-specific procedural effects cannot be fully excluded. Differences in reflux, tract-dependent deposition, local mechanical stress, needle- or catheter-associated tissue responses, and pressure-field dynamics may have contributed to the observed differences in CD3^+^ T-cell coverage and antitumor readouts. In addition, the present study did not include tumor-bearing CED-vehicle controls, pressure-matched sham infusion controls, real-time infusion-pressure monitoring, quantitative reflux assessment, three-dimensional reconstruction, or Vd/Vi analysis. Therefore, our findings should be interpreted as a matched-parameter comparison showing improved early radial CD3^+^ T-cell coverage after CED-TILs, rather than definitive proof that all distribution and efficacy differences were attributable solely to the intrinsic pressure-field advantage of CED.

Safety is a critical consideration for intracerebral cellular therapy. In our short-term safety cohort, naïve rats receiving intracerebral CED-TILs did not show overt neuronal ultrastructural injury on transmission electron microscopy, major hematologic abnormalities, body-weight loss, organ-weight changes, or sensorimotor deficits under the current infusion parameters. These observations suggest that the procedure and cell product were acutely tolerated in non-tumor-bearing rats. However, these findings do not establish long-term safety. First, the safety assessment was performed in non-tumor-bearing rats, whereas tumor-bearing brains may have edema, BBB disruption, necrosis, altered interstitial pressure, and inflammatory activation. Second, the observation window was short and may not detect delayed neuroinflammation, cytokine-mediated toxicity, immune-cell overactivation, edema, off-target tissue injury, or chronic tissue responses. Third, the current study did not evaluate repeated infusions, and the immunogenicity and cumulative toxicity of repeated intracerebral TIL administration remain unknown. Therefore, additional long-term and tumor-bearing safety studies are required before clinical translation can be considered.

The present study has several additional limitations. First, the C6 rat glioma model does not fully reproduce the molecular heterogeneity, infiltrative growth pattern, and immune complexity of human glioblastoma. Although C6 is useful for evaluating intracranial delivery and early therapeutic responses, validation in additional immunocompetent glioma models and patient-derived systems would strengthen translational relevance. Second, the current efficacy experiments used a single intracranial TIL dose, and tissue-based analyses were performed within an early post-treatment window. Thus, we did not determine long-term TIL persistence, trafficking, spatial redistribution, exhaustion state, differentiation status, or functional evolution over time. Third, Ki67 is not tumor-cell specific, and future studies should include tumor-cell markers or lineage tracing to distinguish reduced tumor proliferation from changes in proliferating immune or stromal cells. Fourth, the in vitro RTCA and co-culture assays were performed using technical replicate wells from the same expanded TIL preparation rather than multiple independent TIL expansion batches; therefore, batch-to-batch variability in TIL phenotype, viability, cytokine production, and cytotoxic function was not systematically assessed. Fifth, we tested a single dose, volume, infusion rate, and treatment schedule; dose-response studies and repeated-infusion studies are needed to define the therapeutic window.

From a translational perspective, the current findings should not be interpreted as evidence that CED-TILs are ready for rapid clinical implementation. Rather, they provide an early proof-of-concept framework for stepwise preclinical development of CED-enabled intracranial cellular therapy. First, dose-finding studies should define the optimal TIL number, cell concentration, infusion volume, flow rate, dwell time, catheter geometry, and reflux-control strategy. Second, repeated-dose studies should evaluate TIL persistence, trafficking, functional state over time, immunogenicity, cumulative neuroinflammation, edema, and off-target tissue injury. Third, combination studies should determine how CED-TILs interact with standard-of-care treatments, including radiotherapy, temozolomide, corticosteroid exposure, immune checkpoint blockade, cytokine support, and other immune modulators. Fourth, the feasibility of CED-TILs should be tested in larger brain models using image-guided infusion, real-time distribution monitoring, pressure monitoring, and clinically relevant catheter systems. Finally, CED-TILs should be considered in relation to other BBB-bypass or locoregional delivery strategies, as different approaches may offer distinct advantages depending on tumor location, invasiveness, and therapeutic payload.

In summary, our study supports CED-TILs as an early proof-of-concept locoregional delivery strategy that improves early intracerebral CD3^+^ T-cell coverage and is associated with antitumor activity in an orthotopic rat glioma model. Compared with Control-TILs, CED-TILs enhanced early spatial coverage of CD3^+^ cells, reduced tumor proliferation, increased apoptosis-associated signaling, suppressed tumor progression, and prolonged survival under the tested single-dose conditions. While these results are encouraging, further optimization of the TIL product, infusion parameters, dosing schedule, repeated-infusion safety, long-term persistence, tumor-bearing safety, spatial distribution quantification, and combination strategies will be essential before CED-enabled cellular immunotherapy can be advanced toward clinical application in glioblastoma.

## Data Availability

The original contributions presented in this study are included in the article and Supplementary Materials. The source data supporting the main figures and representative raw data files have been uploaded as Supplementary Materials. Further inquiries can be directed to the corresponding author(s).

## Supplementary Materials

The following supporting information can be downloaded at: https://www.mdpi.com/article/10.3390/biomedicines14071466/s1, Figure S1: CED platform, surgical workflow, experimental timeline, and supplementary quality-control/safety assessments; Table S1: Experimental cohorts, endpoints, timelines, and sample sizes.
