# Supplementary material for: Convection-Enhanced Delivery of Tumor-Infiltrating Lymphocytes Enhances Intratumoral Distribution and Therapeutic Efficacy in an Orthotopic Rat Glioma Model
Source: Biomedicines. 2026 Jun 28;14(7):1466. doi: 10.3390/biomedicines14071466 (PMC13403505; doi:10.3390/biomedicines14071466)
Supplement: Supplementary file 1 [file biomedicines-14-01466-s001.zip › biomedicines-4197769-supplementary.pdf]

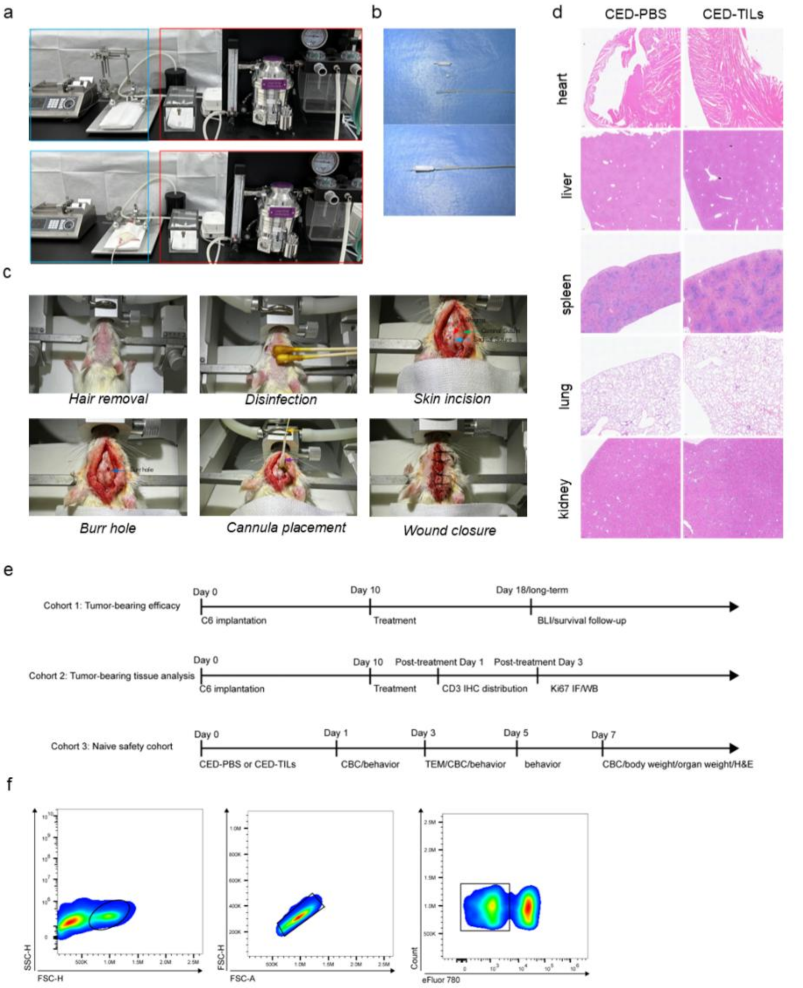

Supplementary Figure S1. CED platform, surgical workflow, experimental timeline, and supplementary quality-control/safety assessments.

a, Representative images of the convection-enhanced delivery (CED) system used for intracranial infusion, including the stereotaxic frame, micro-infusion pump, infusion tubing, catheter connection, and pressure/flow monitoring setup. b, Representative images of the 27G brain infusion catheter used for catheter-based intracerebral CED. c, Representative stepwise surgical workflow for intracranial catheter placement and infusion, including hair removal, disinfection, skin incision, burr-hole drilling, cannula placement, and wound closure. d, Representative hematoxylin and eosin (H&E)-stained sections of major organs from naïve rats receiving CED-PBS or CED-TILs, including heart, liver, spleen, lung, and kidney. e, Schematic timeline of the experimental cohorts. Cohort 1 was used for tumor-bearing efficacy assessment, including C6 glioma implantation on Day 0, treatment on Day 10, and longitudinal bioluminescence imaging (BLI) and survival follow-up from Day 18 onward. Cohort 2 was used for tumor-bearing tissue analysis, including C6 glioma implantation on Day 0, treatment

on Day 10, CD3 immunohistochemistry (IHC) distribution analysis on post-treatment Day 1, and Ki67 immunofluorescence (IF) and western blotting (WB) on post-treatment Day 3. Cohort 3 was used for naïve safety assessment, including intracerebral CED-PBS or CED-TILs on Day 0, complete blood count (CBC) and behavioral testing on Day 1, transmission electron microscopy (TEM), CBC, and behavioral testing on Day 3, behavioral testing on Day 5, and CBC, body weight, organ weight, and H&E analysis on Day 7. f, Representative flow-cytometry quality-control gating plots for expanded rat TILs, including debris exclusion based on FSC/SSC, singlet gating using FSC-A/FSC-H, and live/dead discrimination using Fixable Viability Dye eFluor 780. Live cells were defined as eFluor 780-low events before downstream T-cell phenotype analysis.

Supplementary Table S1. Experimental cohorts, endpoints, timelines, and sample sizes.

Cohort	Purpose / endpoint	Model	Groups	n per group	Intervention / parameters	Time points	Main readouts
Cohort 1	Tumor-bearing efficacy and survival cohort	Orthotopic C6 glioma-bearing rats	NC; Control-TILs; CED-TILs	8 animals/group	C6 implantation on Day 0. Treatment on Day 10. Control-TILs and CED-TILs received 1×10^7 TILs in 20 µL PBS at 2 µL/min, followed by a 5-min dwell period before slow withdrawal.	Day 0: C6 implantation; Day 10: treatment; Day 18 onward: BLI and survival follow-up until humane endpoint	Longitudinal BLI; Kaplan-Meier overall survival; clinical monitoring. Overall survival was predefined as the primary efficacy endpoint.

Cohort	Purpose / endpoint	Model	Groups	n per group	Intervention / parameters	Time points	Main readouts
Cohort 2	Tumor-bearing tissue-distribution and biological analysis cohort	Orthotopic C6 glioma-bearing rats	NC; Control-TILs; CED-TILs	3 animals/group for each tissue endpoint	Same tumor implantation and treatment parameters as Cohort 1. Control-TILs and CED-TILs were matched for TIL dose, infusion volume, infusion rate, stereotaxic target, dwell time, and withdrawal procedure.	Day 0: C6 implantation; Day 10: treatment; post-treatment Day 1: CD3 IHC distribution; post-treatment Day 3: Ki67 IF and WB	CD3 IHC distribution analysis using serial coronal sections and predefined ROIs at 0, 2, and 4 mm from the infusion/needle tract; Ki67 IF in tumor-region ROIs; WB analysis of Ki67, cleaved PARP, and cleaved caspase-3.
Cohort 3	Naïve short-term safety cohort	Naïve non-tumor-bearing rats	CED-PBS; CED-TILs	8 animals/group	Intracerebral CED-PBS or CED-TILs on Day 0. CED-TILs received 1×10^7	Day 0: CED-PBS or CED-TILs; Day 1: CBC and behavior; Day 3: TEM,	Whisker-evoked forelimb placing test; cylinder test; CBC; body weight;

Cohort	Purpose / endpoint	Model	Groups	n per group	Intervention / parameters	Time points	Main readouts
					TILs in 20 µL PBS at 2 µL/min, followed by a 5-min dwell period before slow catheter withdrawal. CED-PBS animals received the same volume and infusion procedure.	CBC, and behavior; Day 5: behavior; Day 7: CBC, body weight, organ weight, and H&E	organ weights; H&E staining of heart, liver, spleen, lung, and kidney; TEM of peri-infusion cortex.
